# Adherence Predictors in Internet-Delivered Self-Help Intervention for Life Stressors-Related Adjustment Disorder

**DOI:** 10.3389/fpsyt.2020.00137

**Published:** 2020-03-13

**Authors:** Evaldas Kazlauskas, Jonas Eimontas, Miranda Olff, Paulina Zelviene, Gerhard Andersson

**Affiliations:** ^1^Center for Psychotraumatology, Institute of Psychology, Vilnius University, Vilnius, Lithuania; ^2^Amsterdam UMC, University of Amsterdam, Amsterdam, Netherlands; ^3^Arq Psychotrauma Expert Group, Diemen, Netherlands; ^4^Department of Behavioural Sciences and Learning, Linköping University, Linköping, Sweden; ^5^Department of Clinical Neuroscience, Karolinska Institute, Stockholm, Sweden

**Keywords:** self-help, adherence, adjustment disorder, internet interventions, treatment

## Abstract

**Background:** There is a growing body of evidence to show that low-intensity self-help internet-delivered interventions are effective in the treatment of mental disorders. Despite the promising effectiveness of internet-delivered interventions, there is still a challenge for mental health services to implement internet-delivered interventions in routine health care. The aim of this study was to analyze the predictors of adherence to a self-help internet-delivered intervention for adjustment disorder.

**Methods:** This was a secondary report of data, including unpublished data, from a randomized controlled trial of an internet-delivered self-help intervention for adjustment disorder. The study included 1,077 participants who had completed online baseline assessments. All participants had experienced significant life stressors over the last 2 years and had high levels of adjustment disorder symptoms. We analyzed the role of sociodemographic variables, pre-treatment adjustment disorder symptoms, outcome expectations, and perceived barriers to mental health services on the use of the intervention.

**Results:** We found that usage of internet-delivered self-help intervention and higher adherence was associated with female gender, greater age, higher pre-intervention outcome expectations, exposure to other forms of psychological therapy in addition to the internet-intervention at the time of the study, and reported perceived barriers to mental health services by the study participants.

**Conclusions:** The findings of the study indicated the importance of non-specific therapeutic factors on adherence during internet-delivered intervention. Perceived barriers to mental health services were associated with higher adherence to self-help intervention, which indicated that communities with restricted access to mental health services could benefit from low-intensity internet-delivered interventions.

## Introduction

Adjustment disorder is among the most commonly diagnosed mental disorders worldwide (1, 2). Until very recently, adjustment disorder received little attention in research (3) and was often viewed as a sub-threshold or mild disorder within the broader spectrum of psychopathology (4). However, adjustment disorder may have a serious effect on a person's functioning following a significant life stressor and is associated with an increased suicide risk (5). The new definition of adjustment disorder given in the 11th edition of the International Classification of Diseases (ICD-11) released by the World Health Organization (WHO) (6) has facilitated research into adjustment disorder (7), including studies on interventions. The ICD-11 defines adjustment disorder as a disorder associated with stress that could be diagnosed as a response to an identifiable stressor with two specific symptoms: (1) preoccupation with the stressor and (2) failure to adapt (6).

It has been recently suggested that low-intensity internet-delivered interventions, including internet-delivered self-guided interventions, could be well-suited for the treatment of adjustment disorder (8). Adjustment disorder is associated with an identifiable stressor, and low-intensity intervention, delivered via the internet or a mobile application, could therefore provide patients with specific coping and stress-management skills targeted at dealing with the stressor-related symptoms. Recent controlled studies have demonstrated that self-guided low-intensity interventions can help to reduce symptoms of adjustment disorder (9–11). Findings from research on adjustment disorder internet intervention are in line with the study outcomes of internet interventions for other mental disorders. There is a considerable body of evidence that shows that internet-delivered interventions (12, 13), as well as internet-delivered self-help interventions, are effective in treating various mental disorders, such as depression, panic attacks, social anxiety, and posttraumatic stress disorder (14–17). However, despite these promising findings, mental health services still find it challenging to implement innovative internet-delivered interventions in routine health care (18).

Exploration of factors contributing to the effectiveness—and understanding the usage patterns—of internet-delivered interventions is important for the development and dissemination of internet-delivered self-help interventions in public health care systems (19). In real-life situations, patients in mental health care services often have comorbid conditions, diverse psychosocial backgrounds, different levels of previous exposure to life stressors, and various symptom levels, and they often come from diverse socioeconomic status groups. Furthermore, users of these interventions have their own expectations regarding the delivery and outcomes of treatment, which may impact their usage of interventions (15). Internet-delivered interventions can reduce barriers to the treatments of mental disorders (20). However, such relatively easier access to interventions via the internet could also be associated with higher attrition rates (21, 22). In contrast to traditional face-to-face treatments where patients need to invest more time and resources into setting up an appointment with a therapist or traveling to a therapy session, internet-delivered interventions can be accessed easier, and patients can more easily select treatment components to engage with or opt out of.

There are many different terms used to refer to participants' activity and their use of internet interventions, such as adherence, attrition, non-usage, and engagement. Several theoretical formulations of adherence that are applicable to internet interventions have been proposed recently (21). However, there is still no common and widely accepted definition or understanding of adherence in internet interventions (23). Despite these ongoing theoretical discussions, recent meta-analytical studies on adherence (24–26) have already identified a number of important predictors of adherence, such as female gender, education level, age, treatment expectations, and baseline symptom severity. The majority of internet-delivered intervention studies have focused on depression or anxiety disorders (12), and, consequently, we know significantly more about adherence predictors from intervention studies of these disorders. Surprisingly, there is a rather small number of studies on internet-delivered interventions for trauma- and stress-related disorders (14, 20, 27). Furthermore, there are only a few internet-delivered intervention studies on adjustment disorder (11, 28–30).

The aim of this exploratory study was to analyze the predictors of adherence and usage activity in self-help internet-delivered intervention for adjustment disorder. We aimed to estimate the role of variables known from previous adherence studies on internet-delivered low-intensity interventions, such as sociodemographic variables, including gender, education level, age, and symptom levels of adjustment disorder. We also evaluated the effects of pre-intervention patient outcome expectations on usage and adherence during an internet-delivered low-intensity self-help intervention, and this was based on previous findings that revealed the importance of expectations in therapy. Furthermore, internet-delivered interventions, especially self-guided interventions, are often regarded as effective low-cost solutions for overcoming barriers to mental disorder treatments, and we explored how perceived barriers to mental health services and previous exposure to traditional face-to-face treatment affects adherence to self-help intervention for adjustment disorder.

## Methods

### Intervention

The intervention was a brief modular internet-delivered CBT self-help intervention for adjustment disorder, and it is described in more detail in the study protocol (28). Access to the intervention website was provided via a computer or a mobile device with an internet connection. The intervention included four modules covering three intervention tasks each, thus totaling 12 tasks. The four modules were body relaxation, mindfulness, time management, and resolving conflicts in interpersonal relationships. Participant activities in the intervention included written tasks, relaxation, and mindfulness practice. The time needed to complete each task ranged from ~5 to 15 min. There was no predetermined sequence of intervention modules, and users of the intervention could choose any task from the list in all modules after logging in to the intervention website (28). After completing the intervention task, participants were asked to evaluate their current stress level, which allowed them to obtain the individualized interactive online chart of stress level changes over time for each participant. Participants received automatic weekly email reminders about participation in intervention after their inclusion in the study.

Data on the efficacy of the intervention revealed medium between-group effect sizes for the primary measures of adjustment disorder symptoms (*d* = 0.57) and the secondary measures of psychological well-being (*d* = 0.51) to the waiting list condition at one-month post-intervention follow-up (9). No additional benefit on primary or secondary outcomes was found in the RCT that evaluated the effects of additional therapist support on the self-help condition in the same intervention (10). Intervention effects *d* ranged from 0.51 to 0.67 in the self-help and self-help with therapist support on-demand group at the one-month follow-up.

### Participants and Procedure

Secondary analyses were conducted using data from a previous controlled trial on a self-help intervention for adjustment disorder (10). Procedures of participant recruitment have been described in greater detail in the trial paper (10). In short, self-referred participants were invited to register for the self-help intervention via social networks and media advertisements. Information about the intervention was made available online via the intervention website. Potential participants were asked to register and provide informed consent before completing the initial online assessment procedure. Inclusion criteria were: (1) ≥18-year-old adults, (2) high levels of adjustment disorder symptoms, (3) exposure to a significant life stressor over the past 2 years, (4) access to a computer or any other device with screen and internet connection, and (5) sufficient Lithuanian language literacy. The study was approved by the Vilnius University Ethics Committee for Psychological Research and was registered in the Australian and New Zealand Clinical Trials Registry with the registration number ACTRN12616000883415.

Out of 1,607 participants who completed the baseline assessment, 530 were excluded for not having met the inclusion criteria—mainly the cut-off score for adjustment disorder symptoms. In total, 1,077 participants who met the inclusion criteria were randomized to two intervention groups: (1) self-help group and (2) self-help group with additional therapist support targeted at aiding the use of the program. The results did not show any significant differences between the two intervention groups as reported in our trial (10). Therefore, we pooled all participants into a single group for further analysis of the usage of intervention. As previously reported, significant intervention effects were achieved after 1 month (9, 10), and baseline and 1-month follow-up data were therefore used in the present analyses.

The demographic characteristics of all study participants are presented in Table 1. Mean age of participants was 35.25 years (*SD* = 11.70), and 81.6% of the participants were female (*n* = 879). Participants were exposed to an average of 4.05 (*SD* = 1.87) life stressors over the last 2 years, mostly in the form of work-related stressors 61% (*n* = 656), conflicts within the family 51% (*n* = 547), and financial difficulties 45% (*n* = 487).

**Table 1:** Characteristics of study participants at baseline.

		**Correlations**									
	**Variable**	***n (%)***	**1**	**2**	**3**	**4**	**5**	**6**	**7**	**8**	**9**
1.	Gender, female	879 (81.6)	–								
2.	Age, mean (SD), range	35.25 (11.70) 18–76	−0.02	–							
3.	Residence, small town or rural	413 (38.3)	0.99	0.20^***^	–						
4.	Education, university degree	771 (71.6)	−0.10^**^	0.21^***^	−0.13^***^	–					
5.	Life stressors over last 2 years, mean (*SD*)	4.05 (1.91)	−0.02	−0.15^***^	−0.02	0.01	–				
6.	Adjustment disorder symptoms (ADNM-8), mean (*SD*)	27.73 (2.78)	−0.07^*^	0.04	0.02	−0.03	0.10^**^	–			
7.	Psychological well-being (WHO-5), mean (*SD*)	36.71 (16.22)	0.00	0.02	−0.04	0.10^**^	−10^**^	−0.39^**^	–		
8.	Barriers to mental health services	314 (29.2)	−0.00	−0.08^**^	0.07^*^	−0.14^**^	0.15^**^	0.11^***^	−0.14^***^	–	
9.	In other psychological therapy at enrolment in the study	180 (16.7)	−0.04	0.07^*^	0.02	0.05	0.01	−0.12^***^	−0.05	−0.11^***^	–
10.	Participants' outcome expectations, mean (*SD*)	2.24 (1.60)	−0.08^**^	0.06	0.01	−0.01	−0.01	0.18^**^	−0.18^**^	0.08^**^	−0.02

*ADNM-8, brief Adjustment Disorder New Module; WHO-5 = World Health Organization Well-being Index*.

* p < 0.05;

** p < 0.01;

*** *p <0.001*.

### Measures

#### Demographic Data and the Use of Psychological Treatments

Gender, university degree, and living in a big city vs. small town/rural area were the main demographic variables used as predictors. Additionally, we asked two questions about participants' experiences of psychosocial treatments. We asked binary questions that required a yes/no response to find out if participants experienced barriers to mental health services and if they were using traditional face-to-face psychological treatment at the start of the internet-delivered intervention.

#### Life-Stressors and Adjustment Disorder

Adjustment disorder symptoms were measured using the brief version of the Adjustment Disorder New Model (ADNM-8) scale (31). The ADNM-8 is comprised of two parts: (1) a 17-item list of life-stressors and (2) an eight-item list of ICD-11 adjustment disorder symptoms. In the first part of the ADNM-8, participants were asked to indicate significant stressors that they had experienced in the last 2 years. In the second part of ADNM-8 participants were asked to rate each of the symptom items on a 4-point Likert type scale (1 = *never*; 2 = *rarely*; 3 = s*ometimes*; and 4 = *often*). The total score of the ADNM-8 is the sum of all item responses. Internal consistency of the symptom part of the ADNM-8 scale was sufficient with Cronbach's alpha = 0.64.

#### Psychological Well-Being

The well-being of participants was measured with the WHO-5 Well-being Index (WHO-5) (32). The WHO-5 is a widely used measure of psychological well-being and is comprised of five items that reflect the presence and absence of positive well-being related to the quality of life (33). Items of WHO-5 are scored on a six-point Likert-type scale ranging from 0 = *at no time* to 5 = *all the time*. The sum of responses is converted to a scale ranging from 0 to 100 by multiplying the raw score by four. A score of 0 indicates the worst well-being, and a score of 100 represents the best possible well-being. Internal consistency for the WHO-5, measured by Cronbach's alpha, was 0.83 in this study.

#### Intervention Outcome Expectations

We used a 10-point Likert-type Subjective Units of Distress Scale (SUD) for the assessment of pre-intervention outcome expectations, following a similar approach as in a recent treatment outcome expectations study (34). Participants were asked to rate their current state at baseline using a single item “*Please, indicate how do you feel today before using BADI program”* on a 10-point SUD scale of natural numbers ranging from 1 = *very bad* to 10 = *very good* presented below the question. Participants were also asked to indicate their expected condition 1 month after the use of the self-help intervention on the same 10-point SUD scale using the item “*Indicate how do you expect to feel after 1 month of usage of the BADI program*”. Participants' outcome expectations were estimated by subtracting the expected SUD score from the current SUD score. A higher score indicated more optimistic outcome expectations, and the scores ranged from −5 to 9 in this study.

### Data Analysis

A multivariate binary logistic analysis with all study variables included in the model was used to predict the usage of intervention to deal with the potential overfitting of the model (35). Data were analyzed using a two-step data analysis plan. Firstly, we searched for predictors of the completion of at least one module of intervention. This was necessary because a significant proportion of randomized participants did not use the self-help intervention after the enrollment. Secondly, we searched for the predictors of engaged participants who completed more than a median (≥4) of intervention tasks of the self-help intervention among those who started to use the intervention after randomization. Additionally, we estimated the predictors of usage activity by adjusting odds ratios (OR) to changes of adjustment disorder symptoms and psychological well-being measured at the one-month follow-up. All statistical analyses were performed using Statistical Package for the Social Sciences IBM SPSS version 24.0.

## Results

One-third of all recruited participants 34.0% (*n* = 366) became intervention users and started using the intervention after the enrollment and randomization. Intervention usage was coded if a participant completed at least one intervention task in one of the modules during the first month since the inclusion in the study. The average number of tasks completed in 1 month of intervention per user was 4.85 (*SD* = 6.87; range 1–57) median = 3. Around a third of users, 34.2% (*n* = 125), completed ≥4 tasks of intervention and were considered high users of intervention. Post-intervention assessment data were available from 105 active users who completed 1-month follow-up assessments.

Descriptive statistics of study variables and correlations among these variables are presented in Table 1. Around one-third of participants (29.2%) reported perceived barriers to mental health services in a community. In total, 16.7 (*n* = 180) participants received other psychosocial treatment at the time of intervention.

### Predictors of Intervention Usage vs. Non-usage

Binary logistic regression analysis (*R*^2^_Nagelkerke_ = 0.044) revealed that female gender (*OR* = 1.43, *p* = 0.046), greater age (*OR* = 1.03, *p* = < 0.001), exposure to traditional face-to-face therapy at baseline (*OR* = 1.65, *p* = 0.004), and higher intervention outcome expectations (*OR* = 1.12, *p* = 0.011) were significant predictors of non-usage vs. usage of the internet intervention measured by at least one completed module (see Table 2). Life stressors, adjustment disorder symptoms or well-being, did not predict the use of the intervention. Moreover, additional therapist support was not a significant predictor of non-usage vs. usage of the intervention after randomization.

**Table 2:** Multivariate binary logistic analysis of self-help intervention adherence predictors.

	**Baseline variables**	**Usage vs. non-usage**	**High usage vs. low usage^a^^,^^b^**	
		***OR* (95% *CI*)**	***OR* (95% *CI*)**	**Adjusted *OR* (95% *CI*)^**c**^**
1.	Gender, female	1.43 (1.01–2.04)^*^	1.63 (0.81–3.26)	1.71 (0.32–9.19)
2.	Age, mean (SD), range	1.03 (1.02–1.04)^***^	1.05 (1.03–1.07)^***^	1.07 (1.02–1.12)^**^
3.	Residence, small town or rural	1.08 (0.82–1.42)	1.06 (0.65–1.71)	0.67 (0.23–1.91)
4.	Education, university degree	0.86 (0.64–1.16)	0.91 (0.53–1.55)	0.25 (0.07–0.93)^*^
5.	Life stressors over last 2 years, mean (*SD*)	1.02 (0.95–1.09)	0.97 (0.86–1.10)	1.06 (0.78–1.44)
6.	Adjustment disorder symptoms (ADNM-8), mean (*SD*)	0.99 (0.94–1.04)	0.96 (0.87–1.04)	0.78 (0.63–0.96)^*^
7.	Psychological well-being (WHO-5), mean (*SD*)	1.00 (0.99–1.01)	0.99 (0.98–1.01)	1.00 (0.97–1.04)
8.	Barriers to mental health services	1.28 (0.96–1.72)^†^	1.76 (1.04–2.96)^*^	2.28 (0.74–6.99)
9.	In other psychological therapy at enrolment in the study	1.65 (1.18–2.32)^***^	1.83 (1.04–3.23)^*^	2.21 (0.70–6.98)
10.	Participants' outcome expectations, mean (*SD*)	1.12 (1.03–1.22)^*^	1.09 (0.93–1.27)	1.69 (1.10–2.59)^*^
11.	Therapist support added during intervention	0.94 (0.72–1.12)	1.13 (0.71–1.81)	0.90 (0.34–2.40)

*ADNM-8, brief Adjustment Disorder New Module; WHO-5, World Health Organization Well-being Index; OR, odds ratio; CI, confidence intervals*.

a *Only participants who completed at least one intervention module were included in the analysis*,

b *High usage = completion of ≥4 intervention tasks*,

c *adjusted for well-being and adjustment disorder symptoms change at post-intervention*.

† p < 0.10;

* p < 0.05;

** p < 0.01;

*** *p < 0.001*.

Gender had a significant effect on usage vs. non-usage, χ^2^(2) = 4.51, *p* = 0.034. A higher percentage of women (35.5%, *n* = 312) started using intervention in contrast to that of men (27.3%, *n* = 54). Participants who actively started using intervention were older (*M* = 37.58, *SD* = 12.62) in comparison to non-users (*M* = 34.05, *SD* = 11.01), *t*_(655.741)_ = 4.74, *p* < 0.001. Intervention outcome expectations among users (*M* = 2.41, *SD* = 1.53) were higher than the non-users (*M* = 2.10, *SD* = 1.59), *t*_(1075)_ = 3.09, *p* = 0.002. Being in another therapy at the start of intervention had a significant effect on the use of intervention, χ^2^(2) = 7.93, *p* = 0.004. A higher percentage of the users of intervention were also undergoing another treatment (21.3%, *n* = 78) than that of the non-users (14.3%, *n* = 102).

### Predictors of Low-Usage vs. High-Usage of the Intervention

Furthermore, we explored the predictors of activity in the intervention as measured by higher engagement in the program and completion of ≤ 4 modules. Binary logistic regression analysis (*R*^2^_Nagelkerke_ = 0.141) revealed that significant predictors were age (*OR* = 1.05, *p* < 0.001), barriers to mental services (*OR* = 1.76, *p* = 0.034), and being in another therapy (*OR* = 1.83, *p* = 0.036). Logistic regression (*R*^2^_Nagelkerke_ = 0.141) with adjusted OR's to therapeutic changes of the primary and secondary outcomes at one-month follow-up revealed such significant predictors as age (*OR* = 1.07, *p* = 0.008), education (*OR* = 0.25, *p* = 0.039), baseline adjustment disorder symptoms (*OR* = 0.78, *p* = 0.019), and pre-intervention outcome expectations (*OR* = 1.69, *p* = 0.016) (see Table 2).

## Discussion

Adherence and attrition have often been identified as important issues in internet interventions (21–23, 36–38). This study contributed to this line of research with the analysis of the predictors of adherence in low-intensity self-help intervention for adjustment disorder. Adherence in this intervention was similar to other large-scale self-help interventions tested in naturalistic settings, which found high non-usage and low adherence rates, [e.g., (36)]. Furthermore, adherence issues were reported in the other recent study of internet self-help adjustment disorder intervention conducted in Switzerland with 48% drop-out rates (11).

Despite low adherence and high non-usage, the large sample size of the study allowed us to conduct an analysis of the intervention usage predictors, which revealed that sociodemographic variables, access to other forms of treatments, perceived barriers to mental health services, and higher outcome expectations were significant predictors of use of the intervention. Similar to the previous studies (24–26) we found that age and gender were important predictors of use of the intervention. Greater age was associated with high use of the intervention also after controlling for symptom change. Female gender was associated with usage vs. non-usage, but it did not predict high usage of the intervention in our study. Furthermore, we found no significant effect of education level on adherence in contrast to several other studies, [e.g., (26, 36)]. However, a large proportion of participants of this intervention had a university degree, which could have an impact on our findings.

The role of pre-treatment symptoms on adherence was ambiguous. Adjustment disorder symptoms at baseline were initially not associated with usage vs. non-usage of intervention. However, after adjusting for symptom change, we found that higher adherence was associated with lower levels of adjustment disorder symptoms at baseline. Furthermore, we found that participants who had already received traditional face-to-face therapy were more adherent users of the intervention. This study did not exclude participants engaged in other forms of therapy, and a surprisingly large proportion of participants at baseline “self-blended” participation in the internet-delivered self-help program with their ongoing other therapy. This exposure to another treatment contributed to higher adherence and was in line with the finding of other studies. Models of blended treatments are expected to provide the advantages of both internet-delivered and face-to-face interventions (18). However, a therapy support condition did not have any effect on adherence in this study.

A significant predictor of higher adherence was perceived barriers to mental health services of study participants. eHealth interventions provided via technology using the internet or mobile applications can help overcome treatment barriers (20) and may contribute to the development of mental health services Around a third of participants reported that they registered for this intervention because they could not access psychological treatments in their community. Participants who reported barriers to mental health services were more adherent users of this low-intensity self-help intervention for adjustment disorder. This finding indicates that usage of internet interventions can be associated with a lack of available mental health services in a community, and people might engage more in internet interventions if they are offered in such developing communities.

Another significant predictor was pre-treatment outcome expectations. Non-usage of participants was predicted by significantly lower pre-intervention outcome expectations. Participants who expected their condition to improve used the intervention and were high users after adjusting findings for intervention outcomes. In line with the previous studies, we found that expectations predicted adherence to the internet-delivered intervention (15, 25, 39, 40). Our findings suggest that outcome expectations might be a relevant and useful predictor of drop-out rates in unguided self-help internet interventions. Participants' with more optimistic pre-treatment outcome expectations were more engaged in the intervention, and high usage was associated with better therapeutic outcomes in this intervention (10). The study also indicated that greater outcome expectations, as a non-specific therapeutic factor, is an important predictor of higher adherence in low-intensity self-help internet-delivered interventions, and it should be addressed in research and implementation of internet interventions in healthcare.

Several limitations of this study should be mentioned. The study sample was large, which enabled us to search for various predictors of adherence in this intervention. However, we included only sociodemographic and psychosocial variables in the analysis of adherence. It is possible that a variety of other barriers and facilitators, such as technological usability, perceived utility, and design of intervention, could contribute to adherence in eHealth interventions (38); thus, the role of these factors could be tested in future adherence studies. Moreover, there are ongoing debates about how to define and measure adherence in internet interventions (21, 37). We analyzed the non-usage, low-usage, and high-usage of intervention in our adherence analysis using the self-reported amount of completed tasks in the intervention to register the usage after access to the intervention had been provided to participants. While this approach is often used in internet intervention research, other ways of registering participant online activity in an intervention using such methods as advanced software solutions or using eye movement tracking could give additional information in further studies.

This study did not reach out to participants characterized by non-usage or low-usage. Interviews with the study participants who refused to use intervention or used it only very little could contribute valuable information on subject of the predictors of adherence. Furthermore, our approach to the assessment of expectations in this study had limited psychometric properties. Further studies are needed with more elaborate methods to measure patient outcome expectations in internet-delivered self-help interventions. Finally, this intervention was offered in Lithuania, a country with limited access to mental health services, and this could have also impacted the participants' usage patterns in the study.

Despite these limitations, the study contributed to the growing knowledge about predictors of adherence in low-intensity self-guided internet interventions with findings about the predictors of intervention on adjustment disorder in a new cultural context. In line with previous studies, we demonstrate that female gender, greater age, and higher expectations are important predictors of adherence across various mental disorders in internet-based treatments, including adjustment disorder. Furthermore, this study points out that perceived barriers to mental health services can increase adherence to self-help internet-interventions, and communities with restricted access to mental health services could benefit from such low-intensity internet-interventions.

## Data Availability Statement

The datasets generated for this study are available on request to the corresponding author.

## Ethics Statement

The study was approved by the Vilnius University Research Ethics Committee for Psychology Research. The study was conducted in accordance with the principles of the Declaration of Helsinki as well as the national Lithuanian and international ethical regulations for research with human subjects. All participants provided written informed consent for participation in the study on a secure online website specially designed for this study.

## Author Contributions

EK: principal investigator, study design, data analysis, the concept of the article, and writing the first draft. JE: data collection, contribution to study planning, and significant revisions of the manuscript. MO: interpretation of results and significant revisions of the manuscript. PZ: data collection and significant revisions of the manuscript. GA: interpretation of results and critical revisions of the manuscript.

### Conflict of Interest

The authors declare that the research was conducted in the absence of any commercial or financial relationships that could be construed as a potential conflict of interest. The handling Editor declared a past co-authorship with one of the authors EK.
